# Experimental study on the seismic performance of a cold-formed thin-walled steel–concrete composite column-H steel beam frame

**DOI:** 10.1038/s41598-023-31789-0

**Published:** 2023-03-18

**Authors:** HongJie Hou, ZhiHua Chen, XiuLi Wang

**Affiliations:** 1 School of Civil Engineering, Lanzhou University of Technology, Lanzhou, 730050 China; 2 School of Civil Engineering, TianJin University, Tianjin, 300072 China

**Keywords:** Civil engineering, Engineering

## Abstract

For lightweight steel frame structures consisting of steel H-beams and cold-formed steel columns filled with concrete, seismic performance comparison tests and numerical simulation analyses were performed for bare and infilled frames. The effects of the lightweight wall panels, the axial compression ratio and the wall thickness of the steel sections of the columns on the seismic properties of the structure were investigated. The failure of the bare frame was concentrated in the weld fractures at the beam-column joints. When the wall panels were embedded in the frame, the damage was concentrated at the corners and edges of the wall panels and the connectors. The wall panels significantly improved the initial stiffness of the frame, early energy dissipation and resistance, and the wall panel energy dissipation rate was initially as great as 91%. As the axial compression ratio increased, the resistance of the structure significantly decreased. Under monotonic loading, the resistance on the structure with an axial compression ratio of 0.4 was reduced by nearly 44% compared to the structure without axial compression. Increasing the wall thickness of the steel sections of the columns increased the load-bearing capacity of the structure, but the increase diminished with increasing wall thickness.

## Introduction

With the rapid development of prefabricated buildings, the application of steel structures in residential buildings is increasing, and the envelope structure has received industry attention. The frame structure with lightweight wall panels embedded (that is, infilled frame), consisting of concrete-filled cold-formed steel columns and hot-rolled H-shaped steel beams, is primarily used in low-rise buildings in rural areas with high seismic fortification intensity. Damage to the frame is mostly controlled by the horizontal load. Although the second-order effect under horizontal load increases the damage of a frame, it has little influence on the whole structure of the low-rise building.

Scholars have studied cold-formed thin-walled steel building members^1–5^, including the shear resistance of walls, the bearing capacity of vertical members, the performance of composite floors, the joints of wall frame columns and the floor beam, the connection performance of the self-tapping screw, and the seismic performance of structures, and the results show that these structures have good seismic performance. The performance of cold-formed thin-walled steel columns and composite beams^6–8^ has also been studied, and the corresponding bearing capacity calculation formula has been derived. The fire resistance and compression capacity of composite concrete-filled cold-formed steel columns^9,10^ have been investigated, and the composite columns exhibited greater compression resistance. Scholars have performed much work on the performance of frames with filled walls^11–13^, and the seismic properties of recycled concrete wall-frame structures have been studied, as have those of frame structures with lightweight wall panels. Additionally, shaking table tests of frame structures with external wall panels^14^ have been carried out, and the composite action performance of wall panels and frames^15^ has been studied. The above research has mostly focused on performance at the component level or the seismic performance of cold-formed thin-walled steel buildings, mainly for multi-story and high-rise buildings. There have been few studies on the seismic performance of low-rise prefabricated light steel frames suitable for rural areas. The seismic performance of light steel frames composed of concrete-filled cold-formed thin-walled steel columns and H-shaped steel beams needs further research. Additionally, the influence of embedded lightweight wall panels and their connections on the performance of this kind of light steel frame still need further research.

When the walls are properly arranged, the walls and the frame are joined to resist horizontal loading, which is not only affected by the wall material but also the connections between the walls and the structure. The wall thickness of the steel section of the composite column is small, and thus the overall performance of the bolt-weld connection and the failure mode of the overall structure need to be further researched. The connections between the light wall panels and the steel frame are usually in the form of U-shaped connectors and mortar joints. The resistance of the wall panel connection to the horizontal action of the wall remains to be further investigated.

In this study, experiments and finite element analyses were performed on the frame. This analysis mainly compared and analyzed the bare and infilled frames. The parametric analysis of the bare frame was mainly performed using the finite element method. The influences of the axial compression ratio and the wall thickness of the cold-formed thin-walled steel of the columns on the structural performance were taken into account. The seismic properties of light steel frame structures were obtained, and they provide a reference for the design of assembled light steel structures in rural areas.

## Specimen design

**Figure 1:**
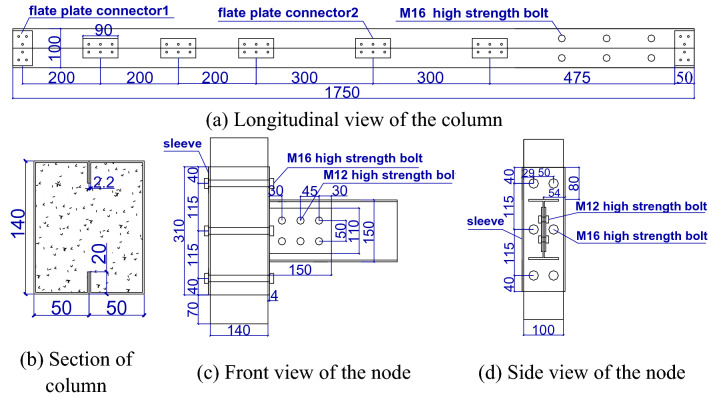
Details of composite columns and joints (unit: mm).

Two single-story single-span 1:2 scale model specimens were designed. The steel was Q235B, and the concrete strength grade was C35. The size and material grade of the finite element model group agreed with those of the test group. The specimens are listed in Table 1. The columns had a rectangular cross section that consisted of two cold-formed thin-walled lipped steel channels. The columns were filled with concrete to avoid local buckling caused by the component’s wide limbs and thin walls. The joints of the columns were connected by flat connectors made of the same material as the columns. The details are shown in Fig. 1a,b. The frame beam adopted hot-rolled steel H-beams that were HN150 × 75 × 5 × 7. The upper joint at the end of each column was in the form of an outer sleeve that was connected to the sleeve by tension bolts, and the sleeve was connected to the H-shaped steel beam by bolt welding, as shown in Fig. 1c,d.

**Table 1:** Sample information.

Test piece grouping	Test piece number	Column section	Beam section	Parametric variable	Loading mode
Test	SF	140 × 100 internal filling concrete	H150 × 75 × 5 × 7	–	Reciprocating loading
	SFW			Embedded wall	
Finite element model	Z0SF	140 × 100 concrete-filled	H150 × 75 × 5 × 7	Axial compression ratio of 0	Reciprocating loading, horizontal monotonic loading
	Z0.2SF			Axial compression ratio of 0.2	
	Z0.4SF			Axial compression ratio of 0.4	
	B2.2SF			Wall thickness of section steel of composite column of − 2.2 mm	
	B4SF			Wall thickness of section steel of composite column of − 4 mm	
	B6SF			Wall thickness of section steel of composite column of − 6 mm

**Figure 2:**
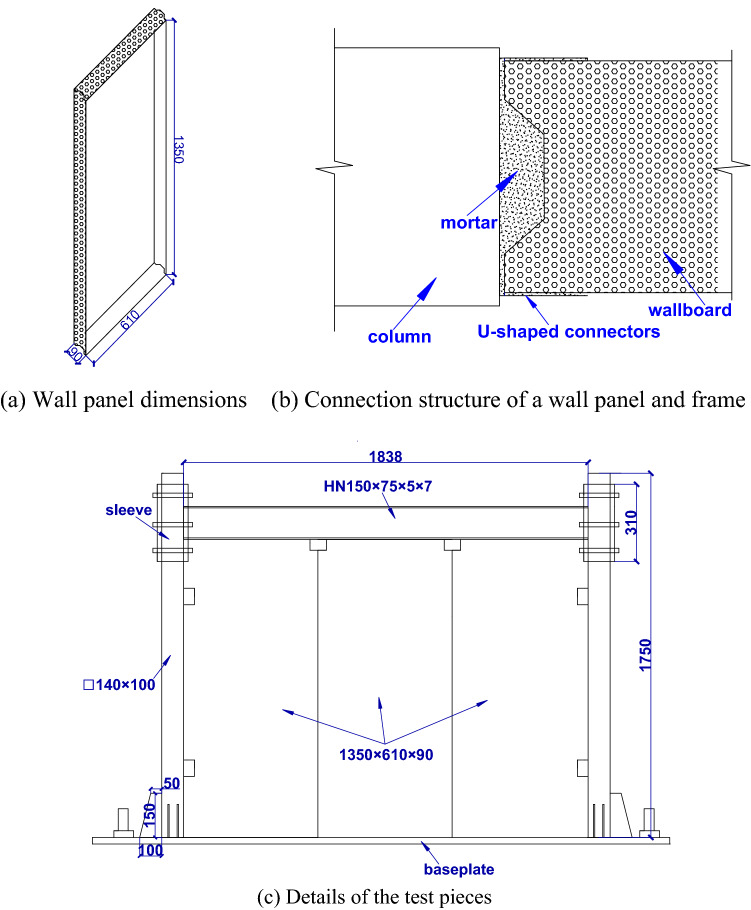
SFW overall diagram (unit: mm).

The wall consisted of three pieces of lightweight wall panels embedded in the frame through U-shaped connectors and cement mortar. A lightweight wall panel is a prefabricated energy-efficient wall that is covered on both sides with calcium silicate slabs and filled with expanded polystyrene (EPS) concrete. The overall details of a SFW specimen, wall size and connection structure are shown in Fig. 2.

## Test loading

### Material test

**Figure 3:**
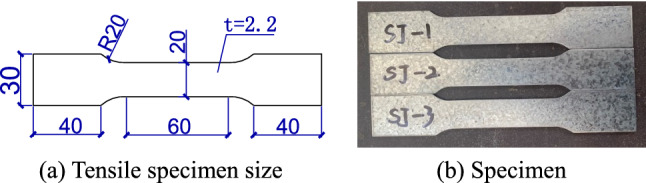
Size and physical specimens of tensile specimens (unit: mm).

According to the provisions of the tensile test for metals^16^, the size of the steel tensile specimens is shown in Fig. 3. The measured properties of the finished material are shown in Table 2. The bolts at the beam-column joints were M12 and M16 high-strength bolts of grade 10.9. The concrete was poured into sections of the columns, and the coarse and fine aggregates are listed in Table 3. Three concrete test blocks were made and cured for 28 days under the same conditions. The measured results for compressive strength^17^ are shown in Table 4. The wall panel had a compressive strength of 3.6 MPa and a Poisson's ratio of 0.21.

**Table 2:** Mechanical properties of finished material.

*f*_y_ (N mm^−2^)	*f*_u_ (N mm^−2^)	*F*_y_/*f*_u_	*E*_s_ (N mm^−2^)	Υ	δ (%)
256.4	307.4	0.83	2.03 × 10^5^	0.33	28.6

**Table 3:** Proportions of concrete mix (unit: kg m^−3^).

Cement	Fly ash	Coarse aggregate	Fine aggregate	Water	Water reducing agent
300	90	1153	621	148.2	4.68

**Table 4:** Properties of concrete.

Test block number	Size (mm^−1^)	Age (day^−1^)	*f*_cu_ (N mm^−2^)
C-1	150 × 150 × 150	28	35.78
C-2			33.44
C-3			36.32

### Test loading scheme and failure criterion

During the test, the loading was stopped when any of the following conditions occurred: (1) the bearing capacity was less than 85% of the ultimate load; (2) excessive weld cracking or bolt cutting occurred; (3) significant local buckling of the beam and column ends or excessive deformation of the beam ends occurred; (4) the corner of the wall panel was crushed or significantly separated from the frame; and (5) there were penetrating cracks in the wall panel.

**Figure 4:**
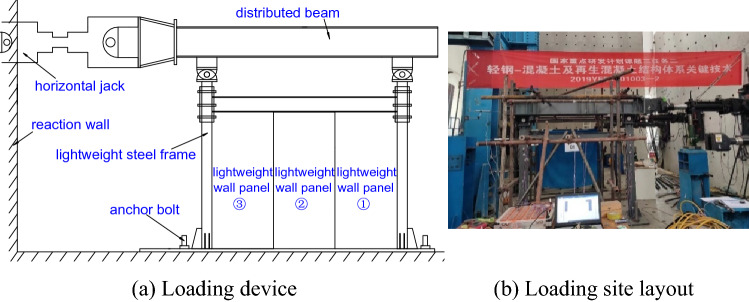
Loading device and site layout.

Due to the limitations of the test site, the MTS actuator applied only horizontal reciprocating loads, as shown in Fig. 4, for the loaded device. The MTS hydraulic servo actuator used in the test was ± 250 mm in stroke, 648 kN in tension and 1013 kN in thrust. The loading rate was 0.5 mm s^−1^, and displacement control loading^15^ was adopted. The specific steps were as follows: (1) when Δ ≤ 8 mm, the displacement increment was 1 mm; (2) when 8 < Δ ≤ 20 mm, the displacement increment was 3 mm; (3) when 20 < Δ ≤ 40 mm, the displacement increment was 5 mm; and (4) when Δ > 40 mm, the displacement increment was 10 mm. The loaded system is shown in Table 5.

**Table 5:** Loading systems.

Load level	Displacement amplitude (mm)	Number of cycles	Load level	Displacement amplitude (mm)	Number of cycles
1	± 1	1	11	± 17	2
2	± 2	1	12	± 20	2
3	± 3	1	13	± 25	2
4	± 4	1	14	± 30	2
5	± 5	1	15	± 35	2
6	± 6	1	16	± 40	2
7	± 7	1	17	± 50	2
8	± 8	1	18	± 60	2
9	± 11	2	19	± 70	2
10	± 14	2	20	± 80	2

### Layout of measuring points

**Figure 5:**
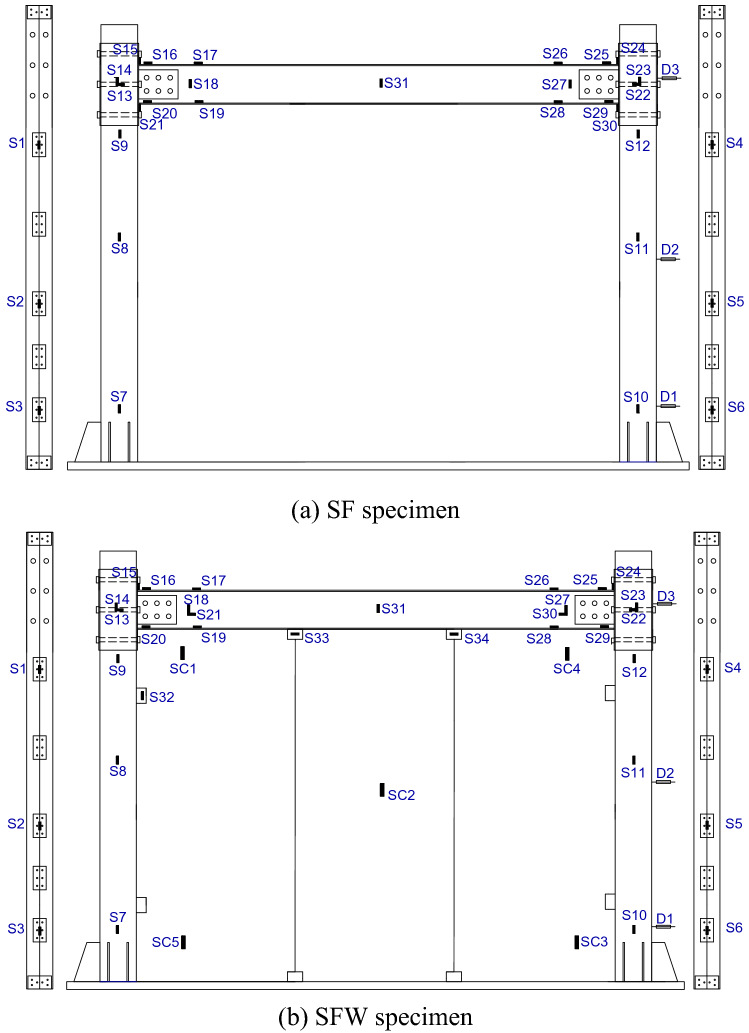
Measurement point arrangement.

Strain data were collected using the DH3816N system with 31 strain gauges pasted on the SF specimen and 38 strain gauges on the SFW specimen. Strain gauges S1–S31 were used to measure the strains on beams, columns and sleeves, S32–S34 for U-shaped connectors, and SC1–SC5 for wall panels. The S1–S12 strain gauges mainly measured the strain of the column at different heights. S31 measured the strain on the beam web at the mid-span, and the remaining strain gauges mainly measured the sleeve strain at the beam-column joints and the strain on the beam. Three displacement meters, D1–D3, were arranged on each specimen, near the foot of the column, in the middle of the column axis and in the middle of the sleeve. The displacement of the column along the loading direction during the test was obtained. The layout of the measurement points of the test specimens is shown in Fig. 5.

## Test results

### Test phenomena and failure characteristics

**Figure 6:**
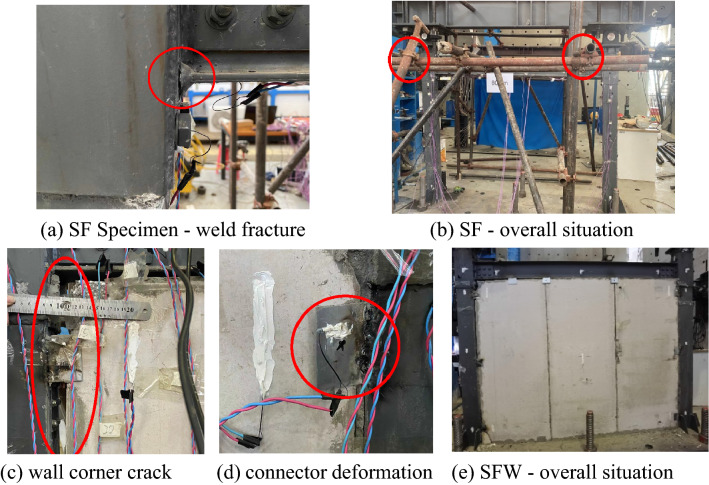
Photographs of specimen failure.

The partial failure of the specimen after test loading is shown in Fig. 6. When loaded to 17 mm, the composite column in the SF specimen made a slight sound, and the stress on the joint of the column began to increase; it rapidly exceeded the yield value of the steel. Tiny cracks appeared in the welds at the joint in the lower flange of the rear beam for loads up to 40 mm. The cracks continued to develop, and crack development at the node was evident when loading was 80 mm. The failure of the SF specimen was concentrated at the beam-column joints, and it was characterized by a break in the weld between the steel beam and the column sleeve at the beam-column joint, with significant deformation of the sleeve joint. At test time, strain variability at the nodes was observed by the data acquisition system. When loaded to approximately 40 mm, the change curves for most measuring points in the acquisition instrument were relatively moderate, and a small number of measuring points still showed a linear upward trend. When tiny cracks appeared in the weld between the beam and the column sleeve, the stress in the welding area where the tiny cracks are produced increased with increasing loading, the widths and lengths of the cracks developed, and more energy was dissipated by crack development. There was no apparent damage to the frame throughout the test, and the structure still showed superior ductility during later stages of loading. When the beam-column joint was joined by a bolt-weld pattern, the weld quality of the joint was somewhat affected due to the small wall thickness of the section steel of the sleeve.

For the SFW specimen, when loaded to 8 mm, the width of the crack in the mortar in the upper part of wall panel ① gradually increased. When loaded to 14 mm, the mortar in the vertical crack spalled. When loaded to 17 mm, the panel of wall panel ③ was partially damaged. When loaded to 25 mm, the gap between the wall and the frame increased, and a significant amount of the mortar filling between them fell away. When loaded to 30 mm, the bottom connector of the wall panel began to deform. When loaded to 40 mm, the upper left corner of wall panel ① was crushed; when loaded to 50 mm, the wall covering panel on the back of wall panel ① was destroyed. When loaded to 70 mm, wall panel ③ separated from the column, and the filling mortar between them entirely fell off. When the load reached 80 mm, the wall panels separated from the frame, the wall panel shifted, and a crack formed in the seam between the wall panels that was approximately 6 mm wide.

### Time dependence

**Figure 7:**
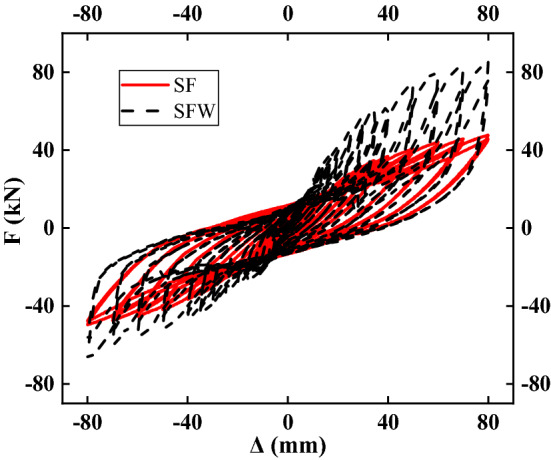
Time dependence of load–displacement curves.

The load‒displacement (P–Δ) curves of the specimens are shown in Fig. 7. In the absence of axial compression, the time record of the SF specimen was tapered, whereas the time record of the SFW specimen had an inverse S-shape, indicating a more pronounced pinching effect. The reason was that in the absence of a vertical load, the slip between the wall panel and the frame was more obvious in the SFW specimen. The SFW specimen exhibited a more obvious positive and negative loading asymmetry than the SF. The main cause of this was the accumulation of damage to the specimen and the looseness of the connecting bolts between the loaded beam and the frame during the test.

As the load increased, the weld line near the joint area of the SF specimen cracked, and then the stress was concentrated in the crack area. The stress in the crack area continued to increase, and the degree of the crack at the place where the microcrack appeared became increasingly severe. There were no new cracks in other parts of the structure. As the small cracks at the joints continued to develop, the residual deformation of the specimen increased, the load–displacement curve of the specimen gradually widened, and more energy was dissipated. The mortar between the frame and the wall panel in the SFW specimen continuously exfoliated. Due to the stiffness mismatch between the frame and the wall panel, the deformation was not coordinated, which resulted in damage first at the weak corner of the wall panel and then along the weak part of the wall panel between the connectors, increasing the degree of damage. Initially, the wall panels and the frame worked together, and the wall dissipated most of the energy. As the damage degree of the wall increased, the connectors gradually were damaged and failed, which weakened the connection between the wall and the frame and decreased the energy consumption of the wall. The equivalent strut effect of the wall during the later loading stage allowed the wall to continue to dissipate energy.

The slope of the curve decreased with loading, and the decreasing trend of the slope was more obvious at later stages of loading, indicating that the residual deformation of the specimen increases and the stiffness of the specimen degraded. In contrast to a bare frame, the frame with infilled walls gradually entered the elastic‒plastic stage and the plastic stage. In the elastic stage, the infilled frame played a good role, and the overall stiffness of the specimen improved. As the loading progressed, the mortar between the wall panel and the frame gradually fell off, and the continuous deformation of the connector weakened the connection effect between the wall and the frame so that the cooperative working performance decreased and the frame energy consumption gradually dominated. In the later stage of loading, although the frame and the wall were separated, the equivalent strut effect of the wall still made the wall play a certain energy dissipation role, but the damage of the wall was significant, and the ability of the frame to resist lateral loading was reduced.

### Skeleton curves

**Figure 8:**
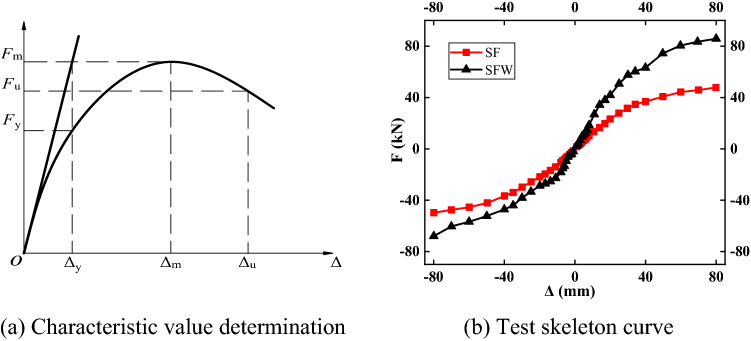
Skeleton curve.

The characteristic values were determined by the method shown in Fig. 8a. The characteristic values are shown in Table 6, and the skeleton curve is shown in Fig. 8b. The skeleton curves of the SF and SFW specimens were double-broken lines. The structure was still able to withstand the load in the event of the weld crack or the separation of the wall panel from the frame, indicating that the structure was resistant to failure. The yield load of the SFW specimen was from 30 to 40% larger than that of the SF specimens, while the increase in resistance was closer to from 79 to 96%, which indicated that the wall had a more obvious effect on the resistance of the structure. The slope of the skeleton curve was larger for the SFW specimen than the SF specimen, which indicated that the initial stiffness of the structure was significantly greater when the lightweight wall panel was embedded in the frame.

**Table 6:** Characteristic points.

Number	Direction	Yield point		Extremum point	
		Δ_y_ mm^−1^	*F*y kN^−1^	Δ_m_ mm^−1^	*F*_m_ kN^−1^
SF	Forward direction	29.59	31.27	79.84	47.79
	Negative direction	− 32.33	− 31.96	− 79.90	− 49.71
SFW	Forward direction	35.61	61.27	79.97	85.82
	Negative direction	− 33.11	− 41.9	− 79.94	− 67.98

The SF specimen was in the elastic deformation stage during the initial loading stage and then entered the plastic development stage. As the loading progressed, cracks continued to develop, and damage accumulated. During the initial loading stage, the mortar of the SFW specimen at the joint between the frame and the wall did not completely fall off, and the frame was in an elastic deformation stage, during which the stiffness of the SFW specimens was larger. The mortar then gradually fell off and peeled off, and a large gap appeared between the wall and the frame, which gradually separated. In the later stages of loading, although the walls were detached from the frame and severely damaged, the equivalent strut formed by the wall panel still played a role and bore part of the horizontal load.

### Stiffness degradation curve

**Figure 9:**
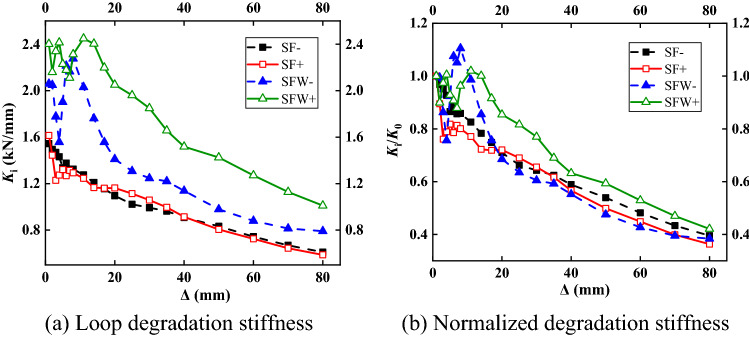
Stiffness degradation.

Figure 9a shows the structural stiffness degradation curve, and the normalized curve is shown in Fig. 9b. The stiffness of the SFW specimen fluctuated while it decreased, increased slightly and then decreased, while the stiffness of the SF specimen continuously decreased. The increase in the stiffness of the SFW was mainly related to the compactness of the connection between the wall panel and the frame. The wall was installed during the winter. The mortar at the joints was affected by the weather, and the wall and frame were not fully filled. When the specimen was stressed, the joint fitted more tightly, and thus the stiffness of the specimen first reduced and then increased. When the gap between the wall and the frame increased, the frame entered the plastic stage, and the stiffness of the SFW specimen decreased.

The faster rate of stiffness reduction in the SFW specimen was due to the larger gap between the infilled wall and the frame and the reduced ability of the two to cooperate. The stiffness of the specimen differed notably under positive loading and negative loading, which was related to the slip caused by the loosening of the loading beam bolts. The initial stiffness of the SFW specimen was from 30 to 50% larger than that of the SF specimen. After loading, the stiffnesses of both specimens decreased to from 35 to 45% of the initial stiffness. The ratios of the final stiffness to the initial stiffness were similar in both specimens, indicating that the structure was mainly determined by the frame at late loading. The effective connection between the wall panel and the frame gradually weakened and disappeared with loading, and the influence of the wall on the seismic properties of the structure gradually diminished due to the aggravation of the local damage of the wall.

### Degradation curve of the bearing capacity

**Figure 10:**
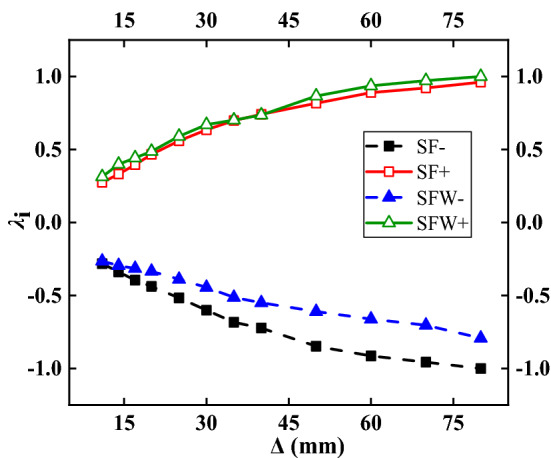
Bearing capacity degradation curve.

The bearing capacity degradation curve is shown in Fig. 10. When Δ > 50 mm, the bearing capacity degradation curve of the SF specimen was relatively flat with little numerical variation, indicating that the bearing capacity of the specimen did not decrease much, and the specimen continued to bear load. When Δ < 50 mm, the degradation coefficient of the bearing capacity of the structure decreased rapidly, and the declining trends of the SF and SFW specimens were basically the same. Under negative loading, the degradation degree of the bearing capacity was smaller in the SFW specimen than in the SF specimen, reflecting the effect of the wall resistance on the horizontal load. Under forward loading, the difference between the bearing capacity degradation curves of the two specimens was small, and the middle parts of the curves overlapped. The main reason was that the wall was badly damaged and detached from the frame, which played a major role at that time. In the later stage of loading, the wall acted as an equivalent strut, but the effect was small because of the severity of its damage.

### Strain

**Figure 11:**
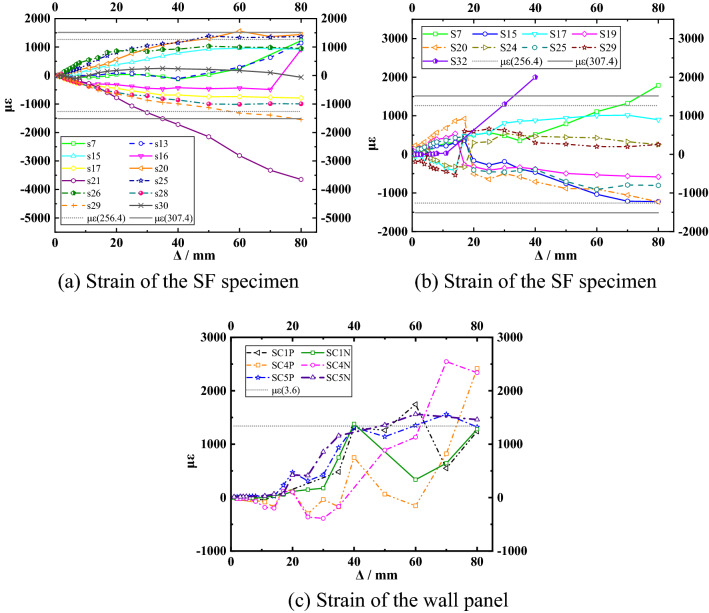
Strain–displacement curves.

The strain diagrams of the partially measured points are shown in Fig. 11. When the loading displacement exceeded 40 mm, the stress at some measurement points of the SF specimen exceeded the yield strength. The measured stress near the column base was close to the yield strength of the steel and should be strengthened in designs. The stress of the frame column developed rapidly from the beginning of loading, but when it reached 40 mm, the stress increased slowly in all but the joint region. After the emergence of a small crack in the node, the node bore a larger horizontal load.

The stresses at the joints of the SFW specimen were within the yield strength, but the stresses at the base of the column were still large. The stress on the U-shaped connector increased after loading to 10 mm, which was related to the shedding of the mortar and the working of the connector. When loaded to 40 mm, the steel stress at the measured point on the connector exceeded its ultimate strength. The connector near the diagonal played a greater role due to the diagonal strut effect of the wall, which also indicated that the connector must be arranged at the diagonal force part of this kind of lightweight wall. In the later stage of loading, the damage was mainly concentrated in the corner of wall panel ① and wall panel ③ and developed along the edge of the wall panel. The strain gauge readings were larger near the corners. It was mainly the equivalent diagonal strut of the wall that acted at this stage, but the damage to the corners was aggravated. The damage to the wall also developed vertically along the corners, and the damage gradually ran through the wall panels between the upper and lower connectors. As the first line of defense during loading, the wall panels continuously dissipated energy and reduced the extent of damage to the frame. As wall damage worsened and the frame carried more load, stress increased and energy was continuously dissipated. At a later stage, the wall still acted as an equivalent strut, but as the damage to the wall corners increased, the point of contact between the wall and the beam-column joints moved downward.

### Energy dissipation capacity

**Figure 12:**
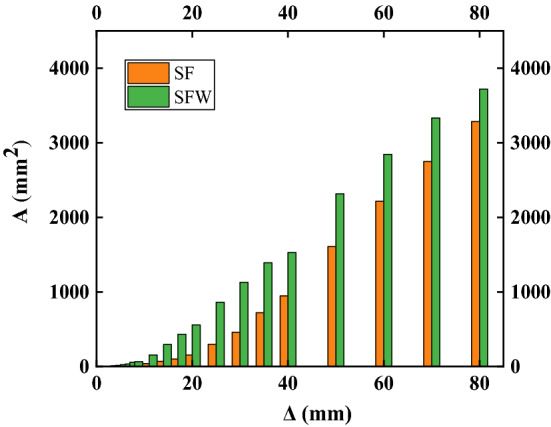
Energy-dissipating histogram.

The energy dissipation capacity of the specimens is shown in Table 7, where E is the energy dissipation coefficient and *ξ*_*e*_ is the equivalent viscous damping coefficient. The energy dissipation column diagram is shown in Fig. 12. The energy dissipation coefficient and the equivalent viscous damping coefficient at the peak were larger for the SF specimen than for the SFW specimen. This indicated that the slip between the wall and the frame was more obvious in the SFW specimen when the wall panel was embedded in the structure. Under the same loading conditions, the plastic development of the frame was slower than that of the SF specimen due to the wall panel embedding, which meant that the SF specimen had greater structural damage. At the initial stage of loading, the equivalent viscous damping coefficient of the SF specimen did not change much. It showed an upward trend with loading, gradually reflecting the development from the elastic stage to the plastic stage. The large value of the equivalent viscous damping coefficient for the SFW specimen at the initial stage of loading suggested that the friction between the wall panel and the connector played a role, but the rapid decrease indicated that the friction effect gradually disappeared. The coefficient gradually increased and then decreased with loading. This change was mainly related to the gradual development of frame plasticity, the gradual disappearance of the effective connection between the wall and the frame, and the aggravation of wall damage. At later times, *ξ*_*e*_ increased more rapidly for the SF specimen than the SFW specimen, which was related to the extent to which the plasticity of the frame developed.

**Table 7:** Specimen energy dissipation.

Specimen	Status	Total energy consumption (kN mm)	ξ_e_	*E*
SF	Peak value	12,708.77	0.134	0.844
SFW		18,772.8	0.096	0.605

Since the wall panels participated in the energy dissipation, the SFW consumed more total energy than the SF specimen. In the early stage, the energy consumption of the SFW specimen was mainly based on the wall and gradually developed into a frame capable of bearing more loads and dissipating more energy. At the beginning, both specimens were in the elastic stage, so the wall energy consumption in the SFW specimen was calculated to be close to 91%, which indicated that the wall acted as the first line of seismic defense. With increasing load, the damage of the wall increased, the joint cracked, the mortar fell off, the frame and the wall gradually separated, and the energy dissipation capacity of the wallboard weakened. At later times, the energy consumption values of the two specimens were not very different, indicating that although the wall dissipated energy through an equivalent diagonal strut, it was the frame that played the main role at this time.

## Finite element parametric analysis

### Model establishment

A model of the lightweight steel frame structure was established by ABAQUS for parameter analysis. The influences of the axial compression ratio and the wall thickness of the section steel of the composite column on the structural properties were investigated in both loading modes. To improve the calculation success rate, the following assumptions were made: (1) the welding quality in the frame structure was reliable. (2) The composite column connection was reliable, and no cracking occurred during the loading process.

The model was established according to the test size. The frame beam, composite columns, connectors, bolts, and so forth in the model were all continuum element C3D8I. Because there were many components in the model that involved contact, constraint and interaction, this element could be better realized and had better calculation accuracy than other options. The constitutive model for steel was a trilinear model with the material data taken from material testing. The constitutive model for concrete was the CDP model, the model developed by Han^18^ was used as the compressive constitutive relationship, and the tensile model of concrete was used as the tensile constitutive relationship^19^. The parameters of the CDP model are given in Table 8. Because of the assumption of the reliable welding quality of the model, a tie constraint was adopted in welding positions, such as beam and sleeve, stiffener and column, column and cover plate. Surface-to-surface contact was used between bolts and column, beams, sleeve, and between concrete and cold-formed thin-walled steel, the tangential behavior was considered along with the penalty, and the normal force was considered along with the hard contact. The friction coefficients were 0.6 between steel and concrete and 0.45 between steel and steel.

**Table 8:** Model parameters of plastic damage of concrete.

Expansion angle	Eccentricity	$f_{b0} /f_{c0}$	$K$	Viscosity coefficient
30	0.1	1.16	0.667	0.005

**Figure 13:**
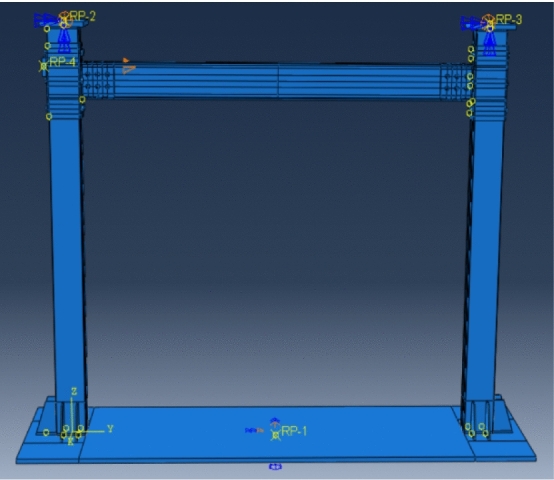
Model and reference points.

The boundary conditions were the same as in the test. For the convenience of imposing boundary conditions and loads, four reference points RP1 to RP4 were set in the model to impose kinematic coupling with a certain surface. The base plate (RP1) was fully consolidated, and the column top (RP2, 2RP3) was subjected to translational constraints in the x-direction and rotational constraints in the y- and z-directions as vertical load application points. Sleeve RP4 applied a reciprocating load. The model and reference points are shown in Fig. 13. The model needed to be segmented into a regular shape, and a global grid of 30 mm was set. Then, the sleeve, beam-column joints and bolt hole were refined.

### Model validation

**Figure 14:**
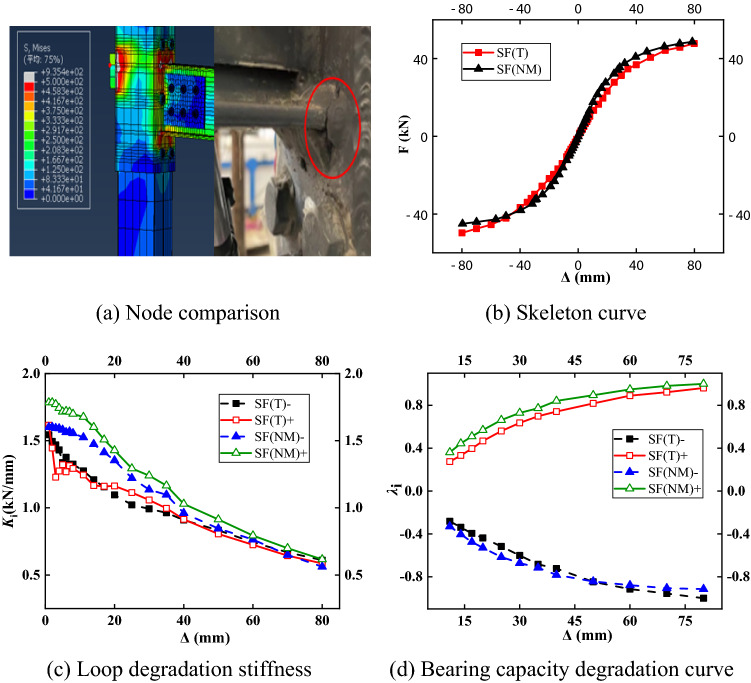
Verification of the finite element model.

Figure 14a–d show the comparison of joints, skeleton curves, bearing capacity, and stiffness degradation curves. In the figure, SF (T) represents the SF test specimen, and SF (NM) represents the SF finite element verification model. The comparison between the test and the finite element results showed that the skeleton curves of the two were in good agreement. Since the finite element model was partially idealized, there were some differences between the two calculations. The resistance of the skeleton curve of the finite element simulation was approximately 10% less than that of the test, the difference in the yield load is less than 4%, and the trend of the curve was consistent. In the finite element model, the structure had a large stress at the joint between the beam flange and the sleeve joint, which was consistent with the phenomenon that the stress at the weld of the joint was too large to break at test time.

### Effect of the axial compression ratio

#### Load–displacement curve

**Figure 15:**
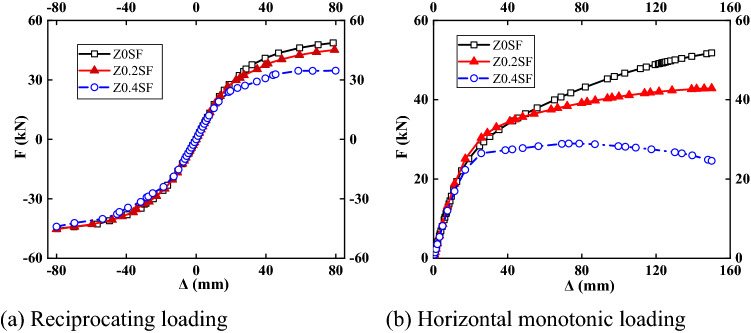
Load–displacement curves.

The model was subjected to cyclic loading and monotonic loading with axial compression ratios of 0, 0.2 and 0.4. The load–displacement curves are shown in Fig. 15. Table 9 lists the characteristic values for different axial compression ratios. For an axial compression ratio of 0.4, the resistance for positive loading decreased significantly, and the resistance for negative loading decreased, but not significantly. The curves for different axial compression ratios before yielding essentially coincided, indicating that the vertical load had little effect on the initial stiffness of the bare frame. When the structure yielded, the structure rapidly entered the plastic stage with an increasing axial compression ratio, which indicated that the greater the axial compression ratio was, the smaller the resistance. In the case of monotonic loading, the resistance of the specimen with an axial compression ratio of 0.4 was reduced by 44% compared to the specimen without axial compression. The failure of the bare frame was mainly caused by the development of plastic at the joints. The higher the axial compression ratio was, the faster the stress developed at the frame joints, the more comprehensive the plastic development at the joints, the faster the frame failed, and the smaller the resistance.

**Table 9:** Characteristic points with different axial compression ratios.

Number	Direction	Yield point		Extremum point	
		Δ_y_ (mm)	*F*y (kN)	Δ_m_ (mm)	*F*_m_ (kN)
Z0SF	Forward direction	27.22	34.22	78.52	48.67
	Negative direction	− 28.77	− 32.34	− 79.52	− 45.04
Z0.2SF	Forward direction	25.13	31.01	79.32	45.13
	Negative direction	− 29.91	− 32.44	− 80	− 45.3
Z0.4SF	Forward direction	19.61	24.43	79.93	34.63
	Negative direction	− 28.41	− 29.26	− 80	− 44.04

#### Stiffness and bearing capacity degradation curves

**Figure 16:**
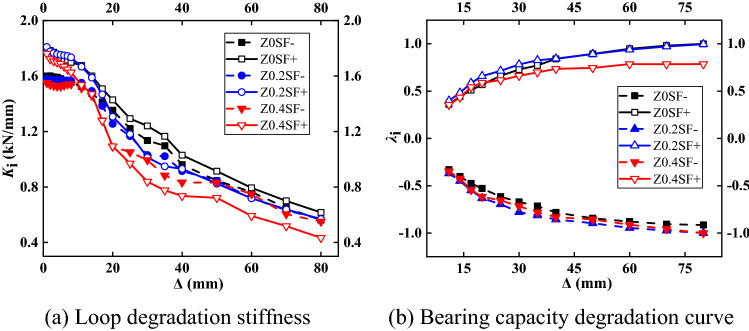
Stiffness and bearing capacity degradation curves.

The curves of structural stiffness and bearing capacity versus axial compression ratio are shown in Fig. 16. The positive initial stiffness of the structure was slightly larger than the negative initial stiffness, and the initial stiffness of the structure decreased slightly as the axial compression ratio increased. The rate of stiffness degradation was faster for an axial compression ratio of 0.4. The trend of the load-bearing capacity degradation curve was consistent. The small axial compression ratio had little effect on the stiffness and bearing capacity of the bare frame. When the axial compression ratio was 0.4, the structure entered the plastic stage more quickly, and the plastic development was faster.

### Effect of the steel wall thickness of the composite column

#### Load–displacement curves

**Figure 17:**
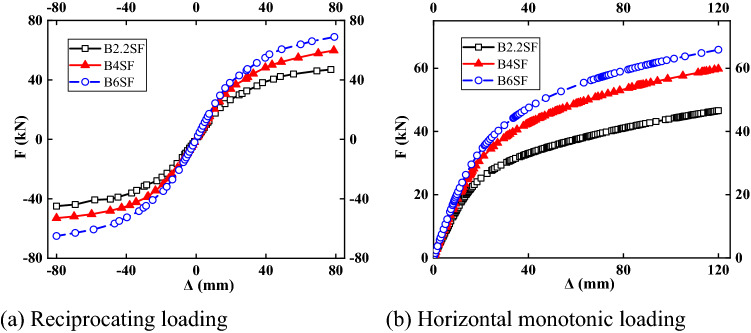
Load–displacement curves.

Table 10 lists the characteristic values for specimens with different wall thicknesses of section steel. Figure 17 shows the load–displacement curves of the specimen under cyclic loading and monotonic loading. Under monotonic load, the load–displacement curves of the structures showed the same trend. However, when the wall thickness of the section steel of the composite column increased to 4 mm, the resistance increased by between 17 and 27%, and when the wall thickness was increased from 4 to 6 mm, the increase range of the resistance decreased. Increasing the section steel wall thickness of the composite column increased the resistance of the structure, but the improvement decreased as the wall thickness increased.

**Table 10:** Characteristic points for different steel wall thicknesses.

Number	Direction	Yield point		Extremum point	
		Δ_y_ (mm)	*F*y (kN)	Δ_m_ (mm)	*F*_m_ (kN)
B2.2SF	Forward direction	26.32	30.82	77.17	46.95
	Negative direction	− 28.39	− 30.66	− 80	− 44.89
B4SF	Forward direction	28.44	40.65	78.89	59.83
	Negative direction	− 28	− 38.86	− 80	− 52.99
B6SF	Forward direction	28.83	46.35	79.18	68.92
	Negative direction	− 30.19	− 46.03	− 80	− 65.02

#### Stiffness and bearing capacity degradation curve

**Figure 18:**
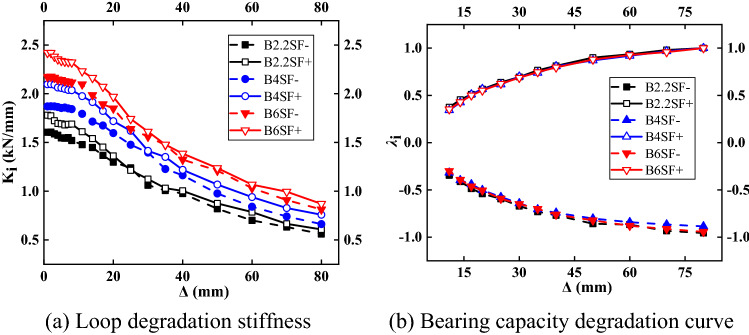
Stiffness and bearing capacity degradation curves.

The curves of the structural stiffness and bearing capacity change with the wall thickness of the section steel are shown in Fig. 18. With increasing wall thickness of the steel section, the initial stiffness of the structure increased. The stiffness degradation trends of different wall thicknesses of steel sections were consistent, but with increasing wall thickness, the speed of stiffness degradation accelerated. The degradation trend lines for the structural bearing capacity essentially coincided, which indicated that increasing the wall thickness of the steel section of the composite column had little effect on the structural bearing capacity.

## Conclusions

In this study, experiments and finite element parametric analyses were conducted to investigate the seismic performance of lightweight steel frames consisting of H-shaped steel beams and thin-walled steel columns filled with concrete. Experiments were performed to analyze the effects of the walls on the initial stiffnesses of the structure and its resistance to horizontal loads. The influences of the axial compression ratio and wall thickness of the steel sections of the columns on the seismic performance of the frames were analyzed using finite element parameterization.

1. When lightweight wall panels were embedded in the steel frame, they had a significant impact on the seismic performance and could improve the resistance and initial stiffness of the structure. The resistance of the structure increased by between 79 and 96%, and the initial stiffness increased by between 30 and 50%. During the later stages of test loading, wall panel failure caused the capacity of the infilled frame to deteriorate faster than that of the bare frame. The effective connection between the lightweight wall panel and frame obviously weakened when the displacement was loaded to 25 mm. When the displacement was loaded to 70 mm, the lightweight wall panels separated from the column.
2. The ductility of the structure was reduced to a certain extent when the wall panel was embedded in the frame. At the initial stage of loading, the wall energy consumption accounted for 91% of the structural energy consumption. The lightweight wall panel was the first line of anti-seismic defense. As wall damage increased, the frame was the main energy dissipation component at late stages of loading. The influence of the lightweight wall panels embedded in the frame on the seismic performance of the structure was nonnegligible.
3. For an axial compression ratio of 0.4, the yield load and resistance of the bare frame were greatly affected, with resistance 44% less than that without axial compression. As the axial compression ratio increased, the stress at the beam-column joints developed rapidly, the plasticity developed rapidly, and the frame failed more quickly. As the wall thickness of the section steel increased, the thickness had little effect on the load-bearing capacity of the structure.

## Data Availability

All data generated or analyzed during this study are included in this published article.
